# High-intensity exercise and cognitive function in cognitively normal older adults: a pilot randomised clinical trial

**DOI:** 10.1186/s13195-021-00774-y

**Published:** 2021-02-01

**Authors:** Belinda M. Brown, Natalie Frost, Stephanie R. Rainey-Smith, James Doecke, Shaun Markovic, Nicole Gordon, Michael Weinborn, Hamid R. Sohrabi, Simon M. Laws, Ralph N. Martins, Kirk I. Erickson, Jeremiah J. Peiffer

**Affiliations:** 1 Discipline of Exercise Science, College of Science, Health, Engineering and Education, Murdoch University, 90 South Street, Murdoch, WA 6150 Australia; 2 Australian Alzheimer’s Research Foundation, Sarich Neuroscience Research Institute, Nedlands, Western Australia Australia; 3 Centre for Healthy Ageing, Murdoch University, Murdoch, Western Australia Australia; 4 School of Medical and Health Sciences, Edith Cowan University, Joondalup, Western Australia Australia; 5 School of Psychological Science, University of Western Australia, Crawley, Western Australia Australia; 6 Australian eHealth Research Centre, CSIRO, Brisbane, Queensland Australia; 7 Department of Biomedical Sciences, Macquarie University, Sydney, New South Wales Australia; 8 Collaborative Genomics and Translation Group, School of Medical and Health Sciences, Edith Cowan University, Joondalup, Western Australia Australia; 9 School of Pharmacy and Biomedical Sciences, Faculty of Health Sciences, Curtin Health Innovation Research Institute, Curtin University, Bentley, Western Australia Australia; 10 Department of Psychology, University of Pittsburgh, Pittsburgh, PA USA

**Keywords:** Exercise, Cognition, Intensity, Genetics, Dementia, Cardiorespiratory fitness

## Abstract

**Background:**

Physical inactivity has been consistently linked to increased risk of cognitive decline; however, studies examining the impact of exercise interventions on cognition have produced inconsistent findings. Some observational studies suggest exercise intensity may be important for inducing cognitive improvements; however, this has yet to be thoroughly examined in older adult cohorts. The objective of the current study was to evaluate the effect of systematically manipulated high-intensity and moderate-intensity exercise interventions on cognition.

**Methods:**

This multi-arm pilot randomised clinical trial investigated the effects of 6 months of high-intensity exercise and moderate-intensity exercise, compared with an inactive control, on cognition. Outcome measures were assessed at pre- (baseline), post- (6 months), and 12 months post-intervention. Ninety-nine cognitively normal men and women (aged 60–80 years) were enrolled from October 2016 to November 2017. Participants that were allocated to an exercise group (i.e. high-intensity or moderate-intensity) engaged in cycle-based exercise two times per week for 6 months. Cognition was assessed using a comprehensive neuropsychological test battery. Cardiorespiratory fitness was evaluated by a graded exercise test.

**Results:**

There was a dose-dependent effect of exercise intensity on cardiorespiratory fitness, whereby the high-intensity group experienced greater increases in fitness than the moderate-intensity and control groups. However, there was no direct effect of exercise on cognition.

**Conclusions:**

We did not observe a direct effect of exercise on cognition. Future work in this field should be appropriately designed and powered to examine factors that may contribute to individual variability in response to intervention.

**Trial registration:**

This study is registered with the Australian New Zealand Clinical Trials Registry (ACTRN12617000643370). Registered on 3 May 2017—retrospectively registered. https://www.anzctr.org.au/Trial/Registration/TrialReview.aspx?id=372780

**Supplementary Information:**

The online version contains supplementary material available at 10.1186/s13195-021-00774-y.

## Introduction

Physical inactivity is considered one of the greatest modifiable risk factors for dementia [1]; however, attempts to provide definitive evidence from randomised controlled trials (RCTs) of a link between exercise and enhanced cognition have been inconsistent. Indeed, a 2015 Cochrane review [2] of RCTs concluded there is insufficient evidence, in cognitively normal older adults, to suggest an effect of exercise on cognition. While a more recent meta-analysis [3], assessing a similar cohort, identified positive effects of exercise when session durations were in excess of 45 min and at least of moderate intensity. These conflicting results indicate the need for greater clarity for the use of exercise as a method for preventing cognitive decline, specifically the precise parameters needed for improving brain health.

When compared with the total volume of physical activity, observational work has reported a stronger association between objectively measured intensity of physical activity and cognitive function [4, 5]. Furthermore, acute bouts of high-intensity exercise improve memory and executive function to a greater extent than moderate-intensity continuous exercise bouts [6, 7]. Although previous work in the area is promising, the importance of exericise intensity in enhancing cognitive health requires rigorous examination in RCTs. A recent 12-week intervention in older adults demonstrated greater improvements in memory after undertaking high-intensity compared with moderate-intensity exercise, or a stretching control [8]. The use of high-intensity exercise is safe in older populations [9] and provides a time-effective method to increase physical health, yet, until more consistent and rigorous evidence is available, the widespread use of high-intensity exercise to enhance cognitive health will continue to be questioned.

Variability across studies might also be explained by factors moderating exercise-induced changes in cognition. Genetic factors, such as the apolipoprotein E (*APOE*) ε4 allele and the brain-derived neurotrophic factor (*BDNF*) Val66Met single nucleotide polymorphism, may modulate the relationship between exercise and brain health [10–12]. The literature in these fields is predominantly sourced from observational studies, which have contributed to largely inconsistent findings. In addition, variability in cognitive response may be due to variability in cardiorespiratory fitness change following exercise [13, 14]. Evidence from RCTs is needed to gain a greater understanding of these potential mediating and moderating effects on cognition.

The current pilot RCT was designed to provide a head-to-head comparison of work-matched moderate-intensity and high-intensity exercise on cognition in cognitively normal older adults. We hypothesised that both intervention groups would receive benefits to cognition, but the high-intensity group would receive additional benefit beyond the moderate-intensity group in a dose-dependent fashion. Based on the hypothesis that increases in cardiorespiratory fitness are an important factor in the relationship between exercise and cognition, we investigated whether change in fitness is associated with improved cognition. Finally, we conducted an exploratory investigation as to whether targeted genetic factors (*APOE* ε4 carriage and *BDNF* Val66Met) moderate the effect of exercise on cognition, and the relationship between altered fitness and cognitive changes. Based on previous literature, we hypothesised that *APOE* ε4 carriers and *BDNF* Val66Met carriers would receive the greatest benefit from exercise, in terms of cognitive improvements [10, 12].

## Methods

### Trial design

The Intense Physical Activity and Cognition (IPAC) study was a single-site parallel pilot randomised controlled trial conducted between October 2016 and September 2019 at Murdoch University and the Australian Alzheimer’s Research Foundation, Western Australia. An open access protocol paper for the IPAC study has been published previously [15]. Participants were randomised to either 6 months of supervised high-intensity exercise, supervised moderate-intensity exercise, or an inactive control group. Reporting of this study adheres to Consolidated Standards of Reporting Trials (CONSORT) guidelines [16].

The IPAC study is registered with the Australian New Zealand Clinical Trials Registry (ACTRN12617000643370). The human research ethics committees at Murdoch University and Edith Cowan University approved the conduct of this study, and all participants provided written informed consent.

### Participants and randomisation

Participants were recruited between October 2016 and November 2017 from a number of sources, including media advertisement, flyers, and word-of-mouth. A full list of inclusion and exclusion criteria have been described previously [15]. Our power analysis was based on the primary outcome of cognitive function: using data from Vidoni et al. [13], we required 28 participants per group to detect differences between three groups at 80% power and the 5% level of significance. A block randomisation protocol (conducted by a researcher who was not collecting outcome data) was used to randomly assign participants to one of the following three groups: high-intensity exercise, moderate-intensity exercise, or a control group.

### Interventions

Participants that were allocated to an exercise group (i.e. high-intensity or moderate-intensity) engaged in exercise two times per week (under the supervision of an Accredited Exercise Physiologist) for 6 months. Each exercise session lasted 50 min and was conducted on a cycle ergometer (Wattbike Pro; Wattbike, Australia). Target intensity was set using the 6 to 20 Borg Scale of Perceived Exertion [17]. Further details of the exercise interventions can be found within the supplementary material.

Adherence to the intervention was measured via session attendance. In addition, exercise intensity was calculated for each participant in the moderate- and high-intensity groups: the percentage of peak aerobic power (measured via a graded exercise test) was calculated for each session (not including the warm-up, cool-down, and for the high-intensity group, the recovery between intervals). The percentage of peak aerobic power in the initial 3 months were calculated using baseline peak aerobic power output, while months 4 to 6 were calculated using peak aerobic power output from the mid-intervention fitness test.

Participants randomised to the control group were provided with an information session regarding the benefits of exercise for overall physical health and known benefits to the brain. Participants in the control group did not receive any exercise instructions.

### Procedures and outcome measures

Full methodological detail regarding outcome measures can be found in the supplementary material.

#### Cognitive assessment

A comprehensive battery of neuropsychological tests was administered to all participants at baseline, 6 months, and 18 months. Composite scores for global cognitive function, attention, episodic memory, and executive function were calculated.

The battery included the Montreal Cognitive Assessment (MoCA), Wechsler Adult Intelligence Scale-III Digit Span, California Verbal Learning Test (CVLT-II), Brief Visual Memory Test (BVMT), Trail Making Test forms A and B, and the NIH EXAMINER Verbal fluency task, Flanker, and Set-shifting. A computerised Cogstate battery (www.cogstate.com) was administered including Groton Maze learning and recall, and identification, detection, one-card learning, and one-back tasks.

We calculated cognitive composite scores using *z*-scores of individual’s performances across all timepoints. For scores where a lower score indicates better performance (i.e. speed), we inversed the score ([score]*-1). The composite scores included the following tasks: (1) Global cognitive composite: Digit Span, Cogstate one-back, Cogstate identification task, CVLT (learning, short delay recall, long delay recall, and recognition d`), BVMT (learning and long delay recall), Cogstate Groton Maze recall, Trails B, Phonemic fluency, Flanker, and Set-shifting; (2) Attention: Digit Span (Forward only) and Cogstate identification task; (3) Episodic Memory: CVLT (short delay recall, long delay recall, and recognition d`), BVMT long delay recall, and Groton Maze recall; and (4) Executive function: Trails B, Phonemic fluency, Flanker, and Set-shifting.

#### Physical assessment

At baseline, 3, 6, and 18 months, all participants underwent a cycling-based graded exercise test to quantify peak aerobic capacity (VO_2_peak) and peak power. All participants also underwent a dual-energy X-ray absorptiometry (DXA) scan, using a Hologic Discovery Bone Densitometer (Hologic, USA), in order to quantify volume of fat, muscle, and bone tissue in the body.

#### Genotyping

TaqMan genotyping assays were performed [18] to determine *APOE* genotype (rs7412, assay ID: C____904973_10; rs429358, assay ID: C___3084793_20) and *BDNF* Val66Met single nucleotide polymorphism (rs6265, assay ID: C__11592758_10). Dichotomous variables indicating *APOE* ε4 carriers or non-carriers and *BDNF* Val66Met carriers or non-carriers were created.

### Statistical methods

Analyses were conducted in R statistical computing packages version 3.6.2 [19] and Statistical Package for the Social Sciences Version 24 (IBM). Data were inspected to determine parametric testing was appropriate for all physiological and cognitive variables.

#### Descriptive statistics

Descriptive statistics were calculated to compare baseline information across study groups. Analyses of variance (for continuous variables) and chi-square analyses (for categorical variables) were conducted to identify differences.

#### Intervention group analysis

All participants that completed a baseline assessment were included in the intention-to-treat (ITT) analyses, regardless of adherence to session attendance, or study withdrawal. To examine the effect of study group on cognition over time, a series of linear mixed models (LMMs) were conducted. Repeated cognitive composite scores were entered as dependent variables, and age, gender, education, time (years), group, and time*group were entered as fixed factors, and participant identification number as a random factor, into the model. Post hoc group comparisons were conducted for any significant time*group interactions (high-intensity as reference group). LMMs were conducted for baseline and 6-month data only, and then again for baseline, 6-, and 18-month data. We report raw mean change scores from baseline and 95% confidence intervals, and unstandardized beta coefficients (B) and their standard error. A positive B represents a positive slope for the moderate/high-intensity groups, compared with the control group.

We ran LMMs to investigate the moderating effects of *BDNF* Val66Met and *APOE* ε4 carriage on cognitive performance over the intervention. Each LMM had an additional interaction of either BDNF*time*group or *APOE* *time*group entered into separate models.

#### Individual variability analysis

The relationships between change in cognition and change in cardiorespiratory fitness (VO_2_peak) from pre- to post-intervention within the high-intensity group only, and for all study participants, were examined. Residualised change scores were generated by entering post-intervention score as the dependent variable and pre-intervention score as the independent variable. Linear models were run with the residualised cognitive change as the dependent variable and residualised fitness change as the independent variable (age, gender, and education as covariates) [20]. For all study participants, the linear models were re-run with the inclusion of either BDNF*fitness change or APOE*fitness change. The cohort was then stratified by *BDNF* Val66Met carriage or *APOE* ε4 carriage and the linear models re-run.

## Results

**Fig. 1:**
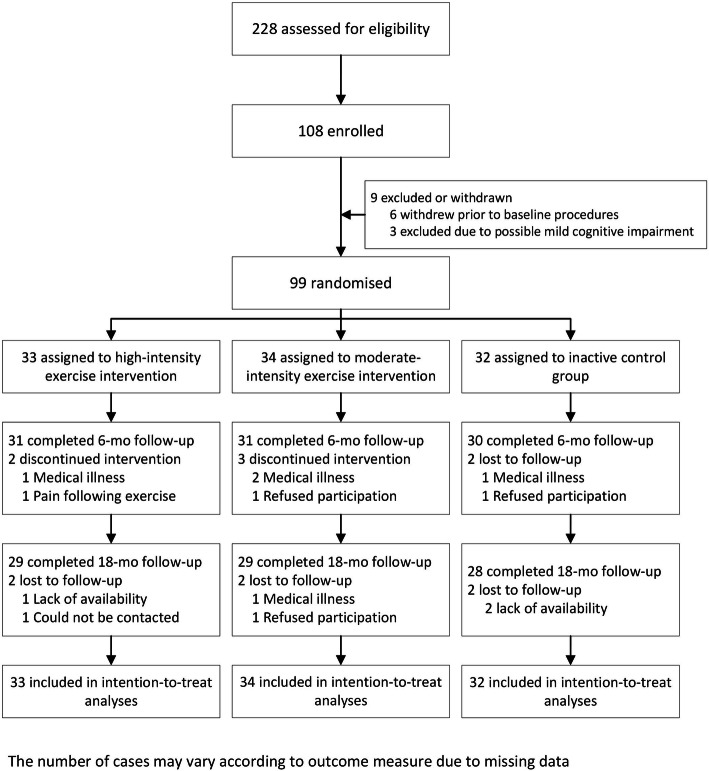
CONSORT diagram for Intense Physical Activity and Cognition study

One hundred and eight participants were enrolled, with ninety-nine completing all baseline assessments and subsequent randomisation to a study group (Fig. 1; descriptive data, Table 1). Seven participants withdrew during the 6-month intervention.

**Table 1:** Descriptive baseline characteristics of study cohort

	High-intensity (*n* = 33)	Moderate-intensity(*n* = 34)	Control (*n* = 32)	Test statistic
**Age,** years	70.2 ± 5.3	68.4 ± 4.2	68.7 ± 5.9	*F* = 1.22
**Gender,** % Female (*n*)	51.5 (17)	52.9 (18)	59.4 (19)	*χ*^*2*^ = 0.79
***APOE*** **ε4 allele carriers,** % (*n*)	27.3 (9)	23.5 (8)	28.1 (9)	*χ*^*2*^ = 0.90
***BDNF*** **Val66Met carriers,** % (*n*)	33.3 (11)	32.4 (11)	50 (16)	*χ*^*2*^ = 2.70
**Years of education**	13.5 ± 2.2	14.2 ± 2.5	14.5 ± 2.1	*F* = 1.65
**Global cognition,** MoCA score	26.0 ± 2.1	26.4 ± 2.8	26.7 ± 2.0	*F* = 0.64
**Baseline VO**_**2**_**peak** (ml/kg/min)	22.2 ± 6.3	24.7 ± 6.9	22.8 ± 6.1	*F* = 1.36
**Baseline peak power** (W)	128.9 ± 49.4	145.0 ± 51.1	126.4 ± 37.1	*F* = 1.57
**Alcohol,** Units per week	5.7 ± 5.9	5.1 ± 5.5	6.4 ± 6.1	*F* = 0.44
**Time from baseline to 6-mo assessment** (wks)	33.0 ± 3.7	34.7 ± 4.7	34.1 ± 2.3	*F* = 1.60
**Time from baseline to 18-mo assessment** (wks)	85.3 ± 3.6	88.3 ± 5.6	87.1 ± 3.9	*F* = 3.38*
**Physical activity** (Met.min/wk^−1^)	4379 ± 3708	4372 ± 2488	3533 ± 1981	*F* = 0.94
**DASS Depression score**	2.3 ± 3.0	1.61 ± 2.1	1.7 ± 1.9	*F* = 0.95
**Daily kilojoule intake**^**a**^	6709 ± 2459	7430 ± 3286	6059 ± 1896	*F* = 2.01
**BMI** (kg/m^2^)	25.8 ± 3.7	26.0 ± 3.9	25.3 ± 3.4	*F* = 0.30
**Waist-hip ratio**	0.87 ± 0.08	0.88 ± 0.07	0.88 ± 0.08	*F* = 0.08

*P < 0.05, post hoc Tukey’s significant difference between high-intensity group and moderate-intensity group. Test statistics determined by one-way analysis of variance for continuous variables and chi-square for categorical variables. Abbreviations: *APOE* apolipoprotein E, *BDNF Val66Met* brain-derived neurotrophic factor Valine66Methionine single nucleotide polymorphism, *BMI* body mass index, *DASS* Depression, Anxiety and Stress Scales, *Met.min/wk*^*−1*^ metabolic minutes per week (subjective habitual physical activity measurement), *MoCA* Montreal Cognitive Assessment, *VO*_*2*_*peak* peak aerobic capacity (fitness measurement), *W* wattage. ^a^Daily kilojoule intake quantified from the Cancer Council of Victoria Food Frequency Questionnaire

### Adherence to prescribed intervention

There was no difference in exercise session attendance between the high-intensity (85.5 ± 12.4%) and moderate-intensity (86.3 ± 9.8%) groups.

The high-intensity group maintained 120.6 ± 25.1% of peak aerobic power during the high-intensity intervals, while the moderate-intensity group cycled continuously at 70.1 ± 16.3% of peak aerobic power.

There were no serious adverse events recorded.

### Group comparisons

A time*group effect was observed for cardiorespiratory fitness, peak aerobic power, and body fat from pre- to post-intervention (Table 2). The high-intensity group experienced greater improvements in cardiorespiratory fitness (+ 24.3%) compared with the moderate-intensity group (+ 12.4%; *B* = 3.92, *p* < 0.01), and control group (+ 2.4%; *B* = 7.36, *p* < 0.001). The high-intensity group also experienced greater improvements in peak aerobic power (+ 29%; *B* = 55.62, *p* < 0.001) and decreases in percentage body fat (− 3.5%; *B* = − 1.59, *p <* 0.05), compared with the control group (peak power change, + 1.3%; percentage body fat change, 0.0%).

**Table 2:** Effects of the exercise interventions on physiological measures and cognitive composite scores

	Raw mean change from baseline (95% CI)			Time*Group (ITT) Unstandardized B (standard error)	
	High-intensity (*n* = 33)	Moderate-intensity (*n* = 34)	Control (*n* = 32)	Baseline to 6 months	All timepoints
**VO**_**2**_**peak (ml/kg/min)**				3.67 (0.72)**	0.35 (0.38)
6	5.40 (4.00, 6.81)	3.02 (1.79, 4.25)	0.55 (− 0.67, 1.77)		
18	0.78 (− 0.62, 2.18)	− 1.43 (− 2.78, − 0.08)	− 0.99 (− 2.85, 0.86)		
**Peak power (Watts)**				27.33 (4.24)**	3.18 (2.51)
6	37.2 (29.4, 45.1)	29.5 (20.6, 38.4)	1.60 (−3.12, 6.33)		
18	7.2 (− 0.6, 15.1)	0.14 (−6.56, 6.85)	− 9.59 (− 16.7, − 2.43)		
**% Body fat**				− 0.80 (0.37)*	− 0.09 (0.23)
6	−1.04 (− 1.83, − 0.25)	− 0.48 (− 1.05, 0.10)	0.00 (− 0.64. 0.63)		
18	1.73 (0.39, 3.07)	2.64 (1.85, 3.44)	1.97 (0.70, 3.24)		
**Global Cognitive composite**				− 0.04 (0.07)	− 0.02 (0.03)
6	0.11 (− 0.03, 0.24)	0.19 (0.09, 0.28)	0.13 (− 0.02, 0.27)		
18	0.18 (0.05, 0.30)	0.21 (0.07, 0.34)	0.23 (0.08, 0.38)		
**Executive function composite**				0.00 (0.09)	0.02 (0.04)
6	0.17 (− 0.00, 0.34)	0.31 (0.14, 0.49)	0.13 (− 0.03, 0.30)		
18	0.30 (0.12, 0.47)	0.31 (0.06, 0.56)	0.18 (0.00, 0.35)		
**Episodic memory composite**				0.02 (0.11)	− 0.04 (− 0.04)
6	0.19 (− 0.04, 0.43)	0.15 (− 0.00, 0.30)	0.13 (− 0.07, 0.34)		
18	0.20 (− 0.04, 0.43)	0.25 (0.07, 0.44)	0.32 (0.12, 0.53)		
**Attention composite**				0.01 (0.15)	− 0.03 (0.05)
6	− 0.17 (− 0.50, 0.15)	0.11 (− 0.12, 0.35)	− 0.22 (− 0.48, 0.04)		
18	− 0.09 (− 0.30, 0.13)	− 0.02 (− 0.26, 0.23)	− 0.10 (− 0.33, 0.13)		

*n* = 99. *p < 0.05, **p < 0.001. Baseline to 6 months is pre- to immediately post-intervention. ‘All timepoints’ includes the full study period of baseline, 6 months, and an 18-month follow-up (i.e. 12 months post-intervention). Abbreviations: *CI* confidence intervals, *ITT* intention-to-treat analyses, *VO*_*2*_*peak* peak aerobic capacity (fitness measurement). All models include age, gender, and years of education as covariates

There were no significant time*group effects on any of the cognitive composite scores. Main effects for time were significant for the executive function composite variable (*p* < 0.05), likely indicating a small practice effect experienced on the tasks assessing this cognitive domain*.* Similarly, there were no significant effects of the genotype*time*group interactions on the cognitive composite scores.

### Individual variability analysis

Within the high-intensity group only, changes in cardiorespiratory fitness were associated with changes in global cognitive function (*F* = 4.91, *p* < 0.05, *η*_p_^2^ = 0.18) and executive function (*F* = 13.89, *p* < 0.001, *η*_p_^2^ = 0.37; Table 3). Increases in cardiorespiratory fitness were associated with improvements in global cognition (*F* = 4.37, *p* < 0.05, *η*_p_^2^ = 0.06) and executive function (*F* = 4.83, *p* < 0.05, *η*_p_^2^ = 0.06) from pre- to post-intervention in the whole sample.

**Table 3:** Changes in cardiorespiratory fitness (residuals) and cognitive function (residuals) from pre- to immediately post-intervention

	Independent variable (***F*** statistic)			
Dependent variable^**a**^	High-intensity group	Whole cohort		
	Fitness change^a^	Fitness change^a^	Fitness change^a^ x *BDNF* Val66Met	Fitness change^a^ x *APOE* ε4 carriage
**Global Cognition change**	4.91*	4.37*	5.13*	2.50
**Executive Function change**	13.89**	4.83*	4.54*	0.33
**Episodic Memory change**	0.68	0.84	4.96*	2.57
**Attention change**	0.94	2.23	0.01	0.13

^a^Residualised change scores created from a linear model where the baseline score was entered as an independent variable and post score (6 months) as the dependent variable. *p < 0.05, **p < 0.001. Covariates: age, gender, years of education. Abbreviations: *APOE* apolipoprotein E, *BDNF Val66Met* brain-derived neurotrophic factor Valine66Methionine single nucleotide polymorphism

**Fig. 2:**
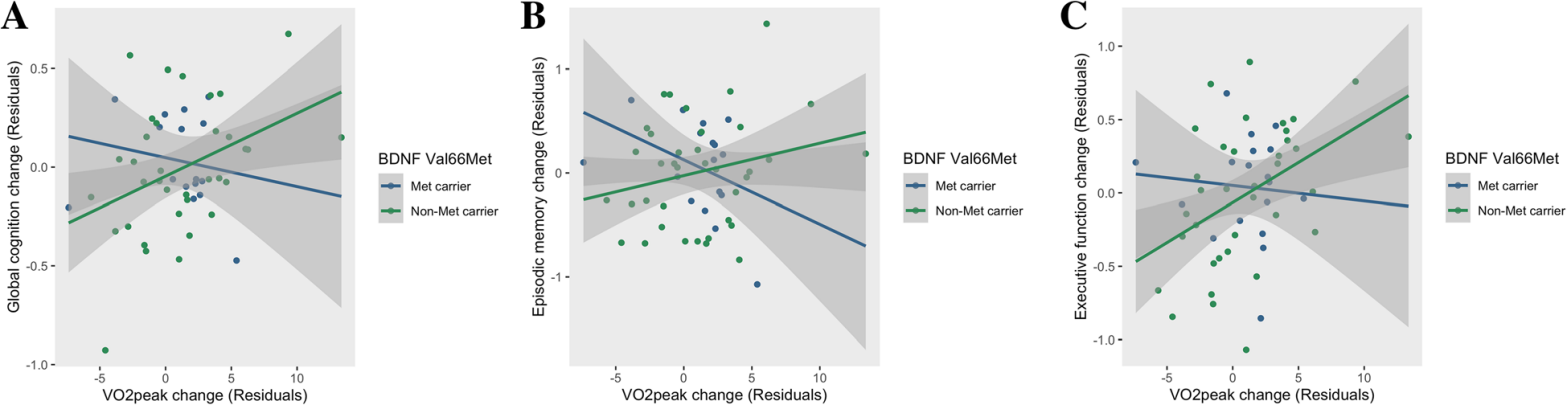
Linear relationships between change in cardiorespiratory fitness (residuals) and change in **a** global cognition (residuals), **b** episodic memory (residuals), and **c** executive function (residuals); from pre- to immediately post-intervention (6 months) in BDNF Val66Met carriers and non-Met carriers. Abbreviations: *BDNF* Val66Met, brain-derived neurotrophic factor Valine66Methionine single nucleotide polymorphism; VO_2_peak, peak aerobic capacity (fitness measurement)

The BDNF*fitness change interaction term was significant for global cognition (*F* = 5.13, *p* < 0.05, *η*_p_^2^ = 0.07), executive function (*F* = 4.54, *p* < 0.05, *η*_p_^2^ = 0.06), and episodic memory (*F* = 4.96, *p* < 0.05, *η*_p_^2^ = 0.07). Post hoc analyses of these interactions revealed non-Met carriers (i.e. *BDNF* Val/Val homozygotes) received benefit in terms of a relationship between change in cardiorespiratory fitness and global cognitive function (*F* = 7.52, *p* < 0.01, *η*_p_^2^ = 0.16) and executive function (*F* = 8.83, *p* < 0.01, *η*_p_^2^ = 0.18; Fig. 2a–c), i.e. greater improvements in cardiorespiratory fitness were associated with greater improvements in cognitive performance post-intervention among non-Met carriers.

We did not observe an effect of APOE*fitness change on any of the cognitive change scores (Table 3). However, following stratification by *APOE* ε4 carriage, only ε4 carriers demonstrated an association between increases in cardiorespiratory fitness and improvements in global cognition (*F* = 4.92, *p* < 0.05, *η*_p_^2^ = 0.23; eFigure 2).

## Discussion

The current study compared the impact of 6 months of supervised high- and moderate-intensity exercise on cognition in a group of cognitively normal older adults. Our intervention successfully delivered high-intensity exercise and resulted in greater increases in cardiorespiratory fitness, compared with the moderate-intensity group. We did not observe any beneficial effects to cognition when comparing group performance from pre- to post-intervention. However, changes in cardiorespiratory fitness from pre- to post-intervention were associated with changes in global cognition and executive function in the high-intensity group, and the entire cohort. From exploratory genetic analyses, we observed moderating effects of the *BDNF* Val66Met polymorphism, whereby the relationship between change in cognition and change in fitness was only evident in Val/Val homozygotes (i.e. non-Met carriers).

When examining the group-level data, we found no effect of either the high- or moderate-intensity exercise interventions on cognitive performance. These findings are inconsistent with a recent RCT which demonstrated improvements on a single high-interference memory task, following a 12-week high-intensity exercise intervention, compared to moderate-intensity exercise [8]. As the cohort within the current study and that investigated by Kovacevic et al. were similar for age, cognitive status, and health, a methodological difference between studies may instead account for the disparate findings. It is possible that more frequent exercise, at least thrice-weekly (delivered by Kovacevic and colleagues), is required to induce cognitive benefit, even with a shorter intervention period (12 weeks). Indeed, the induction of neurotrophic factors (e.g. BDNF) may be required on a more ‘frequent’ basis to contribute to detectable neural benefits [21]. It would not be surprising that duration and frequency, in addition to intensity, play an important role in exercise-induced cognitive response. Thus, future studies are required to further elucidate the optimal exercise parameters for benefiting cognition.

Within the entire cohort, and high-intensity group alone, changes in cardiorespiratory fitness were associated with changes in global cognitive performance and executive function from pre- to post-intervention, with moderate to high effect sizes observed. These data support prior work that has reported relationships between exercise-induced improvements in cardiorespiratory fitness and cognitive changes [13, 20, 22, 23]. The high-intensity exercise intervention in the current study increased cardiorespiratory fitness levels greater than prior similar RCTs in older adults (typically 10–15% increases) [8, 13, 24]. It therefore remains puzzling as to why the observed associations between changes in cardiorespiratory fitness and cognition did not yield group-level differences in cognitive outcomes. While it is possible that individuals with the poorest baseline cognition and fitness levels were more likely to experience fitness-associated cognitive improvement irrespective of exercise intervention, our statistical analysis at least partially corrected for this potential bias. It is also important to consider whether the cognitive assessments used within this generally high-functioning sample were sensitive enough to detect differences between groups.

We conducted an exploratory analysis to examine whether important genetic factors play a role in determining cognitive response following the delivered exercise intervention, and it is important to note that these analyses may have been underpowered. The associations between changes in cardiorespiratory fitness and global cognition, executive function, and episodic memory were strongest in *BDNF* Val/Val homozygotes. Increases in BDNF levels are one of the most well-supported mechanistic theories underlying the relationship between exercise and brain health [25]. BDNF is synthesised in cells as a precursor molecule (pro-BDNF), which undergoes proteolytic cleavage to yield the mature form. Carriage of the *BDNF* Met allele can negatively alter the processing of pro-BDNF to mature BDNF in neurons: validation of our preliminary findings by future work would suggest that exercise may not be potent enough to counteract this detrimental phenotype. Consistent with our findings, a recent systematic review on this topic revealed Val/Val homozygotes are more likely to gain benefit from exercise in terms of better memory performance, compared with Met carriers [26]. Moreover, we also observed a relationship between fitness and global cognition in *APOE* ε4 carriers, but not non-carriers; however, the interaction term APOE*fitness change was not significant (indicating that a moderating effect does not exist). It is likely that combinations of genetic factors influence the relationship between cardiorespiratory fitness and cognition. Previous studies have detected cumulative effects of *APOE* ε4 carriage and *BDNF* Val66Met carriage on cognitive decline [27]; however, our study was not sufficiently powered to examine the APOE*BDNF interaction. Consequently, appropriately powered exercise interventions coupled with hypothesis-driven genetic investigation may reveal more on the ability of exercise to either provide added benefit to those with optimal genetic factors, or alternatively counteract detrimental genotypes.

### Strengths and limitations

The lack of an active control group within the current study contributed to differences in exposure (e.g. social interactions) between our exercise groups and inactive control groups. Nevertheless, as we did not detect group differences across cognitive outcomes, this is unlikely to have affected the results reported here. Our cohort was a generally homogenous sample of highly educated, Caucasian older adults living in the community, and our results may not be applicable to the wider population. Strengths of the study include the three-group design that aimed to detect intensity differences, and our ability to objectively examine intensity levels throughout the intervention. Indeed, our use of rate of perceived exertion to monitor within-subject intensity proved to be an effective method in our older adult cohort, allowing individuals to maintain appropriate intensity targets without the need for frequent testing or the use of monitoring equipment (e.g. heart rate monitors).

## Conclusions

We found that in a cohort of cognitively normal older adults, 6 months of supervised high-intensity and moderate-intensity exercise did not directly contribute to improvements in cognition, compared to an inactive control group. Our data did, however, demonstrate that changes in cardiorespiratory fitness were associated with cognitive change from pre- to post-intervention. Furthermore, our exploratory analysis of important genetic factors provided preliminary evidence that *APOE* and *BDNF* genotypes may affect the relationship between cardiorespiratory fitness and cognitive change. Overall, our data does not provide evidence that high-intensity exercise can contribute to cognitive change in *all* individuals. Future work in this field should be appropriately designed and powered to examine numerous factors that could contribute to individual variability in response to intervention, ultimately leading to individualised prescription of exercise to induce cognitive change and ultimately reduce dementia risk.

## Supplementary Information

**Additional file 1: eFigure 1.** Percentage of peak power for each month of the intervention for the high-intensity and moderate-intensity exercise groups. Each data point represents a participant’s mean peak power (from all attended exercise sessions) for that month. A VO_2_peak test following the third month was used for re-calculation of power. Corresponding months are different between groups (*p* < 0.001); whereby the high-intensity group had higher percentage peak power during all months of the intervention. Abbreviations: M1-6, Month 1 – 6; W, watts. **eFigure 2.** Linear relationship between change in cardiorespiratory fitness (residuals) and change in global cognition (residuals) from pre- to immediately post-intervention (6-months) in APOE ε4 carriers and non-carriers Abbreviations: APOE, Apolipoprotein E; VO_2_peak, peak aerobic capacity (fitness measurement). **eTable 1.** Beta coefficients and standard error of time*group from linear mixed models, and projected sample size for each group to detect a significant effect from longpower function in R statistical package. **Supplementary methods.**

## Data Availability

The datasets generated and/or analysed during the current study are not publicly available due to additional secondary analyses currently being conducted, but are available from the corresponding author on reasonable request.
