# Supplementary material for: High-intensity exercise and cognitive function in cognitively normal older adults: a pilot randomised clinical trial
Source: Alzheimers Res Ther. 2021 Feb 1;13:33. doi: 10.1186/s13195-021-00774-y (PMC7849126; doi:10.1186/s13195-021-00774-y)
Supplement: Supplementary file 1 — Additional file 1: eFigure 1. Percentage of peak power for each month of the intervention for the high-intensity and moderate-intensity exercise groups. Each data point represents a participant’s mean peak power (from all attended exercise sessions) for that month. A VO2peak test following the third month was used for re-calculation of power. Corresponding months are different between groups (p < 0.001); whereby the high-intensity group had higher percentage peak power during all months of the intervention. Abbreviations: M1-6, Month 1 – 6; W, watts. eFigure 2. Linear relationship between change in cardiorespiratory fitness (residuals) and change in global cognition (residuals) from pre- to immediately post-intervention (6-months) in APOE ε4 carriers and non-carriers Abbreviations: APOE, Apolipoprotein E; VO2peak, peak aerobic capacity (fitness measurement). eTable 1. Beta coefficients and standard error of time*group from linear mixed models, and projected sample size for each group to detect a significant effect from longpower function in R statistical package. Supplementary methods. [file 13195_2021_774_MOESM1_ESM.docx]

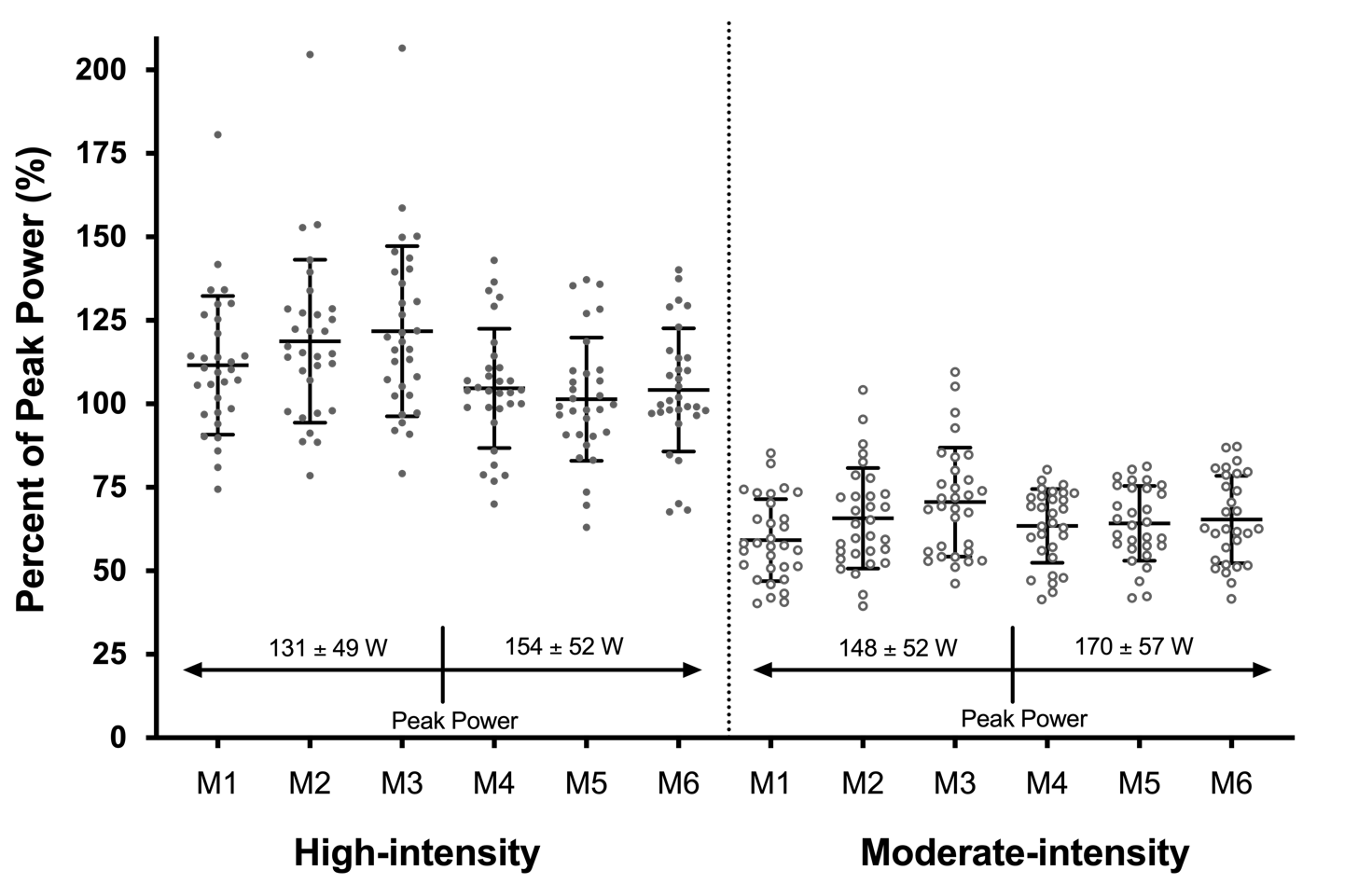


**eFigure 1:** Percentage of peak power for each month of the intervention for the high-intensity and moderate-intensity exercise groups. Each data point represents a participant’s mean peak power (from all attended exercise sessions) for that month. A VO_2_peak test following the third month was used for re-calculation of power. Corresponding months are different between groups (p < 0.001); whereby the high-intensity group had higher percentage peak power during all months of the intervention. Abbreviations: M1-6, Month 1 – 6; W, watts.

***
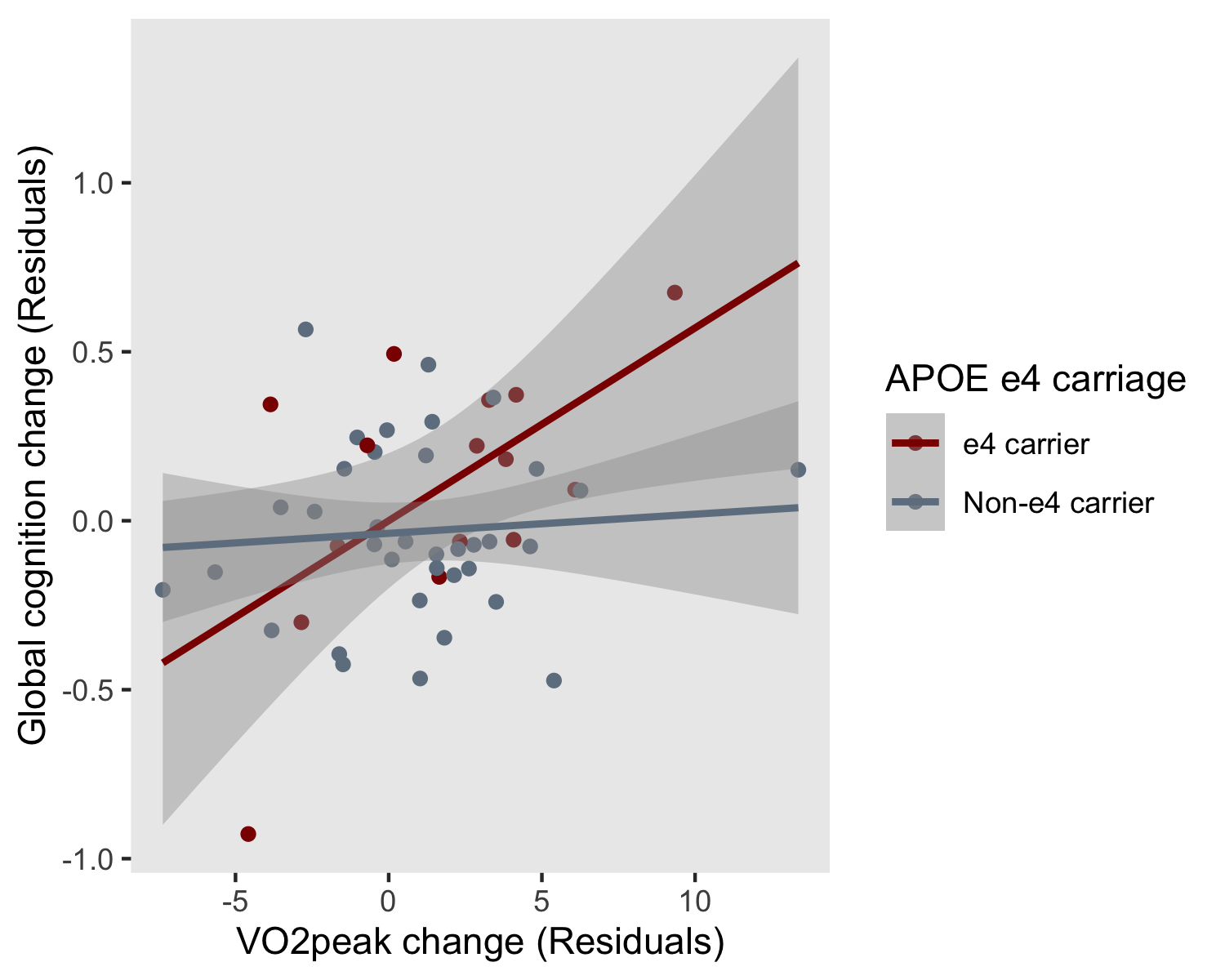
***

**eFigure 2:** Linear relationship between change in cardiorespiratory fitness (residuals) and change in global cognition (residuals) from pre- to immediately post-intervention (6-months) in APOE ε4 carriers and non-carriers Abbreviations: APOE, Apolipoprotein E; VO_2_peak, peak aerobic capacity (fitness measurement)

**eTable 1:** Beta coefficients and standard error of time*group from linear mixed models, and projected sample size for each group to detect a significant effect from longpower function in R statistical package

| **Cognitive composite variable** | **Baseline to 6 months; Intention-to-treat analysis** | | | | | | **All timepoints; Intention-to-treat analysis** | | | | | | |
| --- | --- | --- | --- | --- | --- | --- | --- | --- | --- | --- | --- | --- | --- |
|  | **Control versus High-intensity** | | | **Control versus Moderate-intensity** | | | **Control versus High-intensity** | | | **Control versus Moderate-intensity** | | | |
|  | *B (SE)* | *p-value* | *Projected sample size*  *(per group)* | *B (SE)* | *p-value* | *Projected sample size*  *(per group)* | *B (SE)* | *p-value* | *Projected sample size*  *(per group)* | *B (SE)* | *p-value* | *Projected sample size*  *(per group)* |  |
| **Global Cognitive Composite** | -0.076 (0.136) | 0.58 | 492 | 0.080 (0.134) | 0.55 | 692 | -0.048 (0.052) | 0.35 | 129 | -0.035 (0.050) | 0.48 | 479 |  |
| **Executive Function composite** | 0.010 (0.177) | 0.97 | 782 | 0.216 (0.174) | 0.22 | 625 | 0.042 (0.077) | 0.59 | >1000 | 0.055 (0.076) | 0.47 | >1000 |  |
| **Episodic Memory composite** | 0.035 (0.213) | 0.87 | >1000 | 0.046 (0.205) | 0.82 | >1000 | -0.079 (0.078) | 0.31 | 207 | -0.047 (0.076) | 0.53 | 531 |  |
| **Attention composite** | 0.017 (0.294) | 0.95 | >1000 | 0.492 (0.282) | 0.08 | 123 | -0.053 (0.104) | 0.61 | 420 | 0.001 (0.102) | 0.99 | >1000 |  |

Abbreviations: B (SE), Unstandardised beta (standard error). A positive beta represents a larger positive slope for the moderate/high-intensity groups, compared with the control group.

**Supplementary methods**

**Exercise interventions**

The high-intensity exercise sessions commenced and finished with a 10-minute warm-up and cool-down during which participants cycled at a perceived exertion rating of 11 (30-40% aerobic capacity). The active portion of the intervention involved completion of 11 cycling-based intervals of 1 minute of high exertion (>80% aerobic capacity; 18.0 Borg Scale) interspersed with 2 minutes of active recovery (30-40% aerobic capacity; 12.0 Borg Scale).

The moderate-intensity exercise group participants were required to cycle continuously at a rating of perceived exertion of 13 (50-60% aerobic capacity) for 50 minutes. The high-intensity and moderate-intensity protocols were work-matched based on an 80 kg person with a maximal aerobic capacity of 27 ml.kg^-1^.min^-1^, to yield approximately 386 Met.min^-1^ for a moderate intensity session and 380 Met.min^-1^ for a high-intensity session.

**Graded exercise test**

The graded exercise test followed a step protocol using two-minute stages increasing in intensity until volitional fatigue. The initial power output and step progressions were standardised to baseline body mass to enable similar test durations for individuals: 1) under 70kg, 2) between 70 to 100kg and 3) over 100kg. During the graded exercise test, heart rate was continuously monitored and expired ventilation collected using a Parvo TrueOne metabolic cart (ParvoMedics, USA). VO_2_peak was determined as the highest 15-s mean VO_2_ value recorded in the final 2 minutes of the test.

Peak aerobic power was determined using the following equation: P_LCS_ + F_st_ * BM_P_; where P_LCS_ is the power at the last stage completed, F_st_ is the fraction of the last uncompleted stage and BM_P_ is the body-mass specific increase in work rate per stage.

**Statistical analysis: longpower**

Based on our finding that the exercise interventions did not directly impact cognition, we calculated the post-hoc power that would be required to detect significant differences between groups in each of the cognitive composite scores (using the lmmpower function [23]). Beta coefficients, standard errors, and estimated required samples can be found in eTable1.
